# Low prolactin level identifies hypoactive sexual desire disorder women with a reduced inhibition profile

**DOI:** 10.1007/s40618-023-02101-8

**Published:** 2023-05-18

**Authors:** E. Maseroli, N. Verde, S. Cipriani, G. Rastrelli, C. Alfaroli, S. A. Ravelli, D. Costeniero, R. Scairati, M. Minnetti, F. Petraglia, R. S. Auriemma, R. E. Nappi, M. Maggi, L. Vignozzi

**Affiliations:** 1 Andrology, Women’s Endocrinology and Gender Incongruence Unit, Azienda Ospedaliero-Universitaria Careggi, Florence, Italy; 2 Dipartimento di Medicina Clinica e Chirurgia, Sezione di Endocrinologia, Unità di Andrologia e Medicina della Riproduzione e della Sessualità Maschile e Femminile (FERTISEXCARES), Università Federico II di Napoli, Naples, Italy; 3 Andrology, Women’s Endocrinology and Gender Incongruence Unit, Department of Experimental and Clinical Biomedical Sciences “Mario Serio”, University of Florence, Florence, Italy; 4 Department of Experimental Medicine, Sapienza University of Rome, Policlinico Umberto I Hospital, Rome, Italy; 5 Department of Biomedical, Experimental and Clinical Sciences, Division of Obstetrics and Gynecology, University of Florence, Florence, Italy; 6 Department of Clinical, Surgical, Diagnostic and Pediatric Science, University of Pavia, Pavia, Italy; 7 Endocrinology Unit, Department of Experimental and Clinical Biomedical Sciences “Mario Serio”, University of Florence, Florence, Italy; 8 I.N.B.B. (Istituto Nazionale Biostrutture e Biosistemi), Rome, Italy

**Keywords:** Prolactin, Female sexual dysfunction, Sexual distress, Desire, Hypoprolactinemia

## Abstract

**Purpose:**

Data on the role of prolactin (PRL) in the physiologic range in the female sexual response are scanty. We aimed at investigating the association between PRL and sexual function as assessed by the Female Sexual Function Index (FSFI). We explored the presence of a cut-off level of PRL able to identify Hypoactive Sexual Desire Disorder (HSDD).

**Methods:**

277 pre- and post-menopausal women consulting for Female Sexual Dysfunction (FSD) and sexually active were enrolled in an observational, retrospective study. 42 women were used as no-FSD controls. A clinical, biochemical and psychosexual evaluation was performed. The main outcome measures were: FSFI, Female Sexual Distress Scale-Revised, Middlesex Hospital Questionnaire and Sexual excitation/sexual inhibition scale (SIS/SES).

**Results:**

Normo-PRL FSD women (*n* = 264) showed lower FSFI Desire score than controls (*n* = 42), and higher than hyper-PRL FSD women (*n* = 13). These differences emerged both in pre-menopausal and post-menopausal subjects. In the normo-PRL FSD group, those with PRL in the higher quintile reported higher FSFI Desire scores than those with PRL in the lowest quintile. Women with HSDD presented a lower PRL level than those without (*p* = 0.032). A ROC curve analysis for PRL showed an accuracy of 0.610 ± 0.044 (*p* = 0.014) in predicting HSDD. With a threshold of < 9.83 μg/L, sensitivity and specificity for HSDD were 63% and 56%, respectively. Subjects with PRL < 9.83 μg/L also reported lower sexual inhibition (*p* = 0.006) and lower cortisol levels (*p* = 0.003) than those with PRL >  = 9.83 μg/L.

**Conclusions:**

Hyper-PRL is associated with low desire; however, among normo-PRL FSD women, those with the lowest levels demonstrated a poorer desire than those with the highest levels. PRL < 9.83 μg/L predicted HSDD and a lower sexual inhibitory trait.

**Supplementary Information:**

The online version contains supplementary material available at 10.1007/s40618-023-02101-8.

## Introduction

Prolactin (PRL) is a pituitary hormone, whose release is stimulated by the hypothalamic thyrotropin-releasing hormone (TRH) and inhibited by hypothalamic dopamine [1] through tubero-infundibular, tubero-hypophyseal and periventricular-hypophyseal neurons [2, 3]. The regulation of PRL actions at the central nervous system level occurs via a short and a long feedback loop [4]. A short feedback loop, acting through the hypothalamic neurons, modulates the secretion of PRL itself, while a long feedback loop is responsible for sexual function control acting through the diencephalic neurons, including neurons of the hypothalamus, and specifically of the medial preoptic area (MPOA), the mesolimbocortical (MLC) and the nigrostriatal (NS) neurons [4].

PRL also exerts multiple biological actions on a peripheral level, as confirmed by the widespread and heterogenic expression of its receptors in several tissues [5]. For example, in the mammary gland, PRL is responsible for the lactation process, and is also involved in the regulation of gonadal function in both genders [6–8]. In particular, alterations of either the PRL gene or its receptor have been described as disrupting reproductive function leading to infertility [9, 10]. Beyond its classical actions related to reproductive function, recent evidence strongly suggests that PRL stimulates different brain processes including neurogenesis, neuroprotection, and learning-memory activity, while promoting synaptic plasticity in specific areas of the brain, including those related to sexual function, such as the limbic system [11, 12].

Noteworthy, the physiological role of PRL on the sexual response in humans has been poorly investigated and mainly focused on pathological conditions, enrolling hyperprolactinemic subjects with micro- and macroprolactinomas [13, 14]. Adding layers of complexity, these studies have generated conflicting findings in both genders. In fact, in men, an acute increase in PRL may have physiological inhibiting consequences for sexual function specifically in the post-orgasmic phase, acting through a feedback to dopaminergic neurons [15–17], while hyperprolactinemia is consistently correlated with low sexual desire [13, 18–20]. Of note, Corona et al. first reported that also hypoprolactinemia may exert a detrimental effect on male sexuality, describing an association between PRL levels in the lowest quartile and erectile dysfunction and metabolic alterations on one side, and with premature ejaculation and anxiety symptoms on the other [21]. In women, most studies found that hyperprolactinemia is associated with a multidimensional alteration of sexual function, which could be reverted by normalizing PRL levels [14, 22]. However, two small pilot studies recently indicated that an excessive treatment with dopamine agonists, leading to hypoprolactinemia, induced an impaired sexual function, which was normalized by reducing drug doses in both genders [23, 24].

## Aim

The present study is aimed at investigating the association of PRL level in the normal range with sexual function, and in particular, with the desire domain as assessed by the Female Sexual Function Index (FSFI) [25]. We also explored the presence of a cut-off level of PRL able to differentiate women with and without a diagnosis of Hypoactive Sexual Desire Disorder (HSDD).

## Materials and methods

### Subjects and setting

In this retrospective study, a consecutive series of 277 female patients attending our Andrology, Women’s Endocrinology and Gender Incongruence Unit at Careggi University Hospital, Florence, Italy for sexual symptoms, between January 2016 and January 2021, were analyzed. The inclusion criteria were: being sexually active in the previous 4 weeks, having a partner and being able to give an informed consent. Two-hundred sixty four of these patients constituted the normo-PRL Female Sexual Dysfunction (FSD) women group, while 13 were diagnosed with hyperprolactinemia and formed the hyper-PRL FSD group. The diagnostic workflow for these pathological conditions was performed according to the current Endocrine Society guidelines and the reasons for hyper-PRL were identified as follows: pharmacological treatment (78%), macroprolactinemia (11%) and undefined cause due to loss at follow-up (11%). A third group (*n* = 42) was identified as that composed by healthy controls, namely women consulting the same Unit for other endocrinological symptoms, contraceptive counseling or menopausal check-up, in which non-compensated endocrine disorders had been excluded, sexually active in the previous 4 weeks, and having a partner. For these women, the Female Sexual Function Index (FSFI) and the Female Sexual Distress Scale-Revised had been used as screening tools and were retrospectively available, and the FSFI total score was ≥ 26.55, excluding FSD (see Clinical Assessment).

All procedures were in accordance with the ethical standards and approved by the institutional research committee. All participants provided an informed consent before any diagnostic procedure (protocol 37.589/SPE.13.034, Careggi Hospital, Florence, Italy).

### Clinical assessment

For all patients, a demographic, clinical and anthropometric [weight, body mass index (BMI), waist circumference (WC) and systolic (SBP) and diastolic blood pressure (DBP)] evaluations were performed. Current use of medications, in particular those potentially interfering with PRL levels (psychiatric medications, hormonal contraception, hormonal replacement therapy), was investigated.

All participants were asked to complete the FSFI [25], the most common psychometric instrument for the screening of FSD. The validated Italian version was used [26]. This self-reported questionnaire explores different domains of the female sexual response: desire, arousal, orgasm, satisfaction, and pain. For each question, the possible score ranges from 0 or 1 to 5, with the minimum score representing the most pathological condition. By adding individual questions’ scores and multiplying these by a specific factor, a score for each domain is obtained. The sum of the 6 domain scores provides a Total score, with a threshold of <  = 26.55 classifying the woman as at risk for FSD. The FSFI does not evaluate the presence of distress, which plays a key role in the diagnosis of FSDs, including HSDD. Therefore, we included in our questionnaires also the Female Sexual Distress Scale-Revised (FSDS-R) [27], a 13-items self-reported questionnaire which quantifies sexually related personal distress in women with HSDD. The cut-off score for discriminating affected from non-affected patients is >  = 11.

Psychopathologic parameters were evaluated by the Middlesex Hospital Questionnaire (MHQ), a self-administered measure of psychoneurotic pathology in non-psychiatric settings [28]. The MHQ provides scores for free-floating anxiety (MHQ-A), phobic anxiety (MHQ-P), obsessive–compulsive traits and symptoms (MHQ-O), somatization (MHQ-S), depressive symptoms (MHQ-D), and histrionic or hysterical symptoms and traits (MHQ-H) and a total score. Body image concerns, which represent a crucial aspect in women’s sexuality, were explored by the Body Uneasiness Test (BUT), a validated self-reported questionnaire [29]. This measure includes two separate sheets, one for the evaluation of body experiences (BUT-A), i.e., weight phobia or compulsive self-monitoring, and one regarding dissatisfaction with individual body parts (BUT-B); the answers are scored on a 6-point Likert scale, with higher scores identifying greater concerns or dislike. Both sub-scales provide total indexes.

Finally, FSD patients completed the Sexual Inhibition and Sexual Excitation Scales (SIS/SES) [30, 31]. The SIS/SES is composed of 3 factors, the first (SES) describing sexual arousal related to sexual or non-sexual situations, the second (SIS1) describing inhibition due to threat of performance failure (e.g., arousal difficulties, concern for the partner’s pleasure, etc.), and the third (SIS2) describing inhibition due to threat of performance consequences (e.g., the risk of being caught, sexually transmitted diseases, pain, unwanted pregnancies) [30].

Biochemical assessment was performed in the morning, in the early follicular phase of the cycle for pre-menopausal women—in any day of the month in post-menopausal women- in fasting conditions to measure glucose (esokinase method; Dimension Vista 1500 Medical Solutions by Siemens Healthcare, Newark, USA), total cholesterol, high-density lipoprotein (HDL) and triglycerides (automatic enzymatic colorimetric method; Dimension Vista 1500 Medical Solutions by Siemens Healthcare, Newark, USA); insulin (electrochemiluminescence immunoassay, “ECLIA”; Roche Diagnostics, Mannheim, Germania) and glycated hemoglobin (HbA1c) (high prestation liquid chromatography, HPLC, Variant II method, Biorad Laboratories, Hercules, CA, USA), prolactin (PRL), thyroid-stimulating hormone (TSH), follicle-stimulating hormone (FSH), luteinizing hormone (LH), 17β-estradiol (using the chemiluminescence method; DIMENSION VISTA ® System, Siemens), cortisol (electro-chemiluminescence immunoassay; COBAS 600, Roche Diagnostics, Basel, Switzerland); sex hormone binding globulin (SHBG) (using the electrochemiluminescence immunoassay; COBAS, ROCHE, Germany). Androgens, including D-4-androstenedione, were measured by liquid chromatography-mass spectrometry (Agilent Technologies, Santa Clara, CA). To estimate low-density lipoprotein (LDL) cholesterol the Friedewald equation was used: LDL cholesterol = total cholesterol-(HDL cholesterol + triglycerides/5).

Metabolic syndrome (MetS) was diagnosed according to the National Cholesterol Education Program—Third Adult Treatment Panel (NCEP-ATPIII; Expert Panel on Detection, Evaluation, and Treatment of High Blood Cholesterol in Adults, 2001), based on the presence of ≥ 3 of the following factors: central obesity (waist circumference ≥ 88 cm), hypertriglyceridemia (≥ 150 mg/dL or specific therapy), arterial hypertension (systolic blood pressure ≥ 130 mmHg and/or diastolic blood pressure ≥ 85 mmHg or specific therapy), impaired fasting glycemia (≥ 110 mg/dL or specific therapy) and low HDL cholesterol serum levels.

### Statistical analysis

Data were reported as mean ± SD when normally distributed, as median (quartiles) when non-normally distributed and as percentage and number when categorical. Linear analyses were performed to assess the association of continuous variables (PRL) using the Pearson’s method. Significant correlations at univariate analysis were tested at multivariate analysis after adjusting for confounding factors (i.e., years since menopause). One-way ANOVA was used to test differences in means among groups (i.e., PRL quintiles), followed by post hoc analyses, applying a Bonferroni correction. The unpaired 2-sided Student t-test and the Mann–Whitney *U* test were applied for assessing differences between 2 groups, whenever appropriate. Receiver operating characteristic (ROC) analysis was carried out to determine the PRL cut-off value that yielded optimal sensitivity and specificity in identifying the presence of HSDD.

All analyses were carried out with SPSS 26.0 statistical package and a *p* < 0.05 was considered statistically significant.

## Results

Table 1 shows clinical, biochemical and psychometric parameters of normo-PRL (*n* = 264) and hyper-PRL (*n* = 13) women consulting for FSD, and no-FSD controls (*n* = 42). Statistically significant differences among the 3 groups, and derived from pairwise comparisons, are reported. Partner’s sexual dysfunction (as perceived by the patient) was more common in FSD normo-PRL (32%) than in FSD hyper-PRL women (7.7%). As expected, FSD normo-PRL women showed significantly lower FSFI and higher FSDS-R scores as compared to controls (Table 1). When considering FSFI subdomains, Desire and Satisfaction were the only 2 subdomains in which FSD hyper-PRL women displayed lower scores when compared to FSD normo-PRL women (Table 1). When adjusting the differences between FSD hyper-PRL and FSD normo-PRL women for partner’s sexual dysfunction, graduation and current use of hormonal replacement therapy, only that in Desire retained statistical significance (*F* = 7.172, *p* = 0.008 for Desire; *F* = 0.377, *p* = 0.540 for Satisfaction). The differences in FSFI Desire among the 3 groups were maintained even when data were analyzed according to pre-menopausal vs. post-menopausal status (Supplementary Fig. 1, panels A and C).

**Table 1:** Sociodemographic and clinical characteristics of normoprolactinemic and hyperprolactinemic women consulting for FSD and a control group of normoprolactinemic, no-FSD women

	FSD Normo-PRL (*n* = 264)	FSD Hyper-PRL (*n* = 13)	No FSD (*n* = 42)	*p*
Sociodemographic parameters				
Age (y)	45.6 ± 12.8	44.3 ± 6.7	42.5 ± 12.5	0.204
Graduated, % (*n*)	26.1 (69)°	61.5 (8)*	21.4 (9)	**0.044**
Physical activity, % (*n*)	39.2 (103)	23.1 (3)	42.8 (18)	0.072
Smoking habit, % (*n*)	18.9 (50)	7.7 (1)	19.0 (8)	0.593
Drinking habit, % (*n*)	16.1 (42)	7.7 (1)	11.9 (5)	0.559
Recreational drug use, % (*n*)	3.8 (10)	0.0 (0)	4.7 (2)	0.909
Clinical parameters and comorbidities				
Parity, % (*n*)	55.0 (145)	53.8 (7)	45.2 (19)	0.480
Current use of hormonal contraception, % (*n*)	9.3 (25)	7.7 (1)	7.1 (3)	0.224
History of unwanted sexual experiences, % (*n*)	32.5 (86)	38.4 (5)	NA	0.812
Diabetes mellitus, % (*n*)	6.0 (16)	0.0 (0)	9.5 (4)	0.334
Metabolic Syndrome, % (n)	19.5 (51)	7.7 (1)	23.8 (10)	0.441
Oncologic diseases, % (*n*)	10.2 (27)	23.1 (3)	7.1 (3)	0.298
Neurologic diseases, % (*n*)	2.0 (5)	0.0 (0)	4.8 (2)	0.295
Menopause, % (*n*)	45.6 (120)	53.8 (6)	30.9 (29)	0.085
Years since menopause (y) for menopausal women	3.4 ± 5.5	2.4 ± 4.1	2.0 ± 4.1	0.165
Current use of hormonal replacement therapy in menopausal women, % (*n*)	4.0 (11)°	23.1 (3)*	0.0 (0)	**0.026**
Urinary or gynecologic diseases, % (*n*)	55.9 (148)	30.7 (4)	21.4 (9)	0.512
Body mass index (kg/m^2^)	25.1 ± 5.9	22.6 ± 3.1	25.2 ± 6.2	0.301
Waist circumference (cm)	93.5 ± 15.8	87.4 ± 10.0	94.9 ± 14.8	0.355
Systolic blood pressure (mmHg)	120.0 [110.0 – 135.0]	120.0 [110.0–128.7]	125.0 [110.0–140.0]	0.472
Diastolic blood pressure (mmHg)	75.0 [70.0—80.0]	70.0 [70.0–78.5]	80.0 [68.7–80.0]	0.242
Laboratory parameters				
Prolactin (μg/L)	9.4 [7.2–13.7]°	29.8 [25.9–35.3]	11.3 [7.8–16.2]	** < 0.001**
Fasting glycemia (mmol/L)	4.99 ± 1.11	4.78 ± 0.72	5.03 ± 0.93	0.671
T otal cholesterol (mmol/L)	5.26 ± 1.02	5.59 ± 0.98	5.17 ± 1.11	0.434
HDL cholesterol (mmol/L)	1.65 ± 0.42	1.89 ± 0.52	1.61 ± 0.43	0.172
LDL cholesterol (mmol/L)	3.17 ± 0.88	3.35 ± 0.89	3.08 ± 0.97	0.607
Triglycerides (mmol/L)	0.89 [0.65–1.22]	0.69 [0.60–1.01]	0.97 [0.64–1.25]	0.265
Morning cortisol (nmol/L)	426.6 ± 148.3	558.4 ± 177.8	418.9 ± 158.0	0.051
TSH (mIU/L)	1.8 [1.2–2.5]	2.5 [1.7–2.7]	1.9 [1.1–2.6]	0.149
FSH (IU/L)	18.6 [6.6–72.1]	46.5 [6.3–95.2]	9.5 [6.8–52.6]	0.154
LH (IU/L)	11.6 [5.2–33.4]	20.4 [5.9–42.6]	6.8 [4.7–25.1]	0.093
17b-estradiol (pmol/L)	109.0 [62.2–182.4]	110.0 [65.4–178.1]	150.0 [73.5–316.2]	0.649
Total testosterone (nmol/L)	0.9 [0.5–1.4]	0.8 [0.5–1.4]	0.9 [0.6–1.4]	0.773
D-4-androstenedione (nmol/L)	3.6 [2.1–6.4]	4.6 [2.3–5.9]	4.2 [2.4–4.6]	0.714
DHEA-S (μmol/L)	3.3 ± 2.4	2.6 ± 2.0	3.9 ± 2.5	0.120
SHBG (nmol/L)	65.0 [45.1–91.1]	80.5 [46.8–105.7]	58.0 [41.4–82.7]	0.413
Psychosexual parameters				
History of psychiatric diseases, % (n)	42.1 (111)	38.4 (5)	11.9 (5)	0.055
Current use of psychiatric medications, % (n)	25.5 (67)	53.8 (7)	14.3 (6)	0.118
MHQ total score	36.0 [28.0–47.0]*	46.0 [33.2–54.2]	27.0 [19.0–43.2]	**0.014**
BUT-A GSI (global severity index)	1.0 [0.5–1.5]	0.7 [0.1–2.6]	1.0 [1.0–2.0]	0.630
BUT-B PSDI (positive symptom distress index)	2.2 [1.6–2.8]	2.1 [1.8–3.5]	2.3 [1.7–2.7]	0.373
BUT-B PST (positive symptom total)	5.0 [0.0–13.0]	5.0 [0.0–10.2]	7.0 [0.0–11.5]	0.793
Sexual Inhibition Scale (SIS) 1	36.0 [25.0–38.0]	37.0 [29.0–38.0]	NA	0.343
Sexual Inhibition Scale (SIS) 2	35.0 [30.5–39.5]	36.0 [25.0–41.00]	NA	0.557
Sexual Excitation Scale (SES)	43.0 [33.5–49.5]	47.0 [34.0–58.0]	NA	0.510
Stable relationship, % (n)	92.7 (245)	92.3 (12)	90.5 (38)	0.817
Conflicts within the couple, % (n)	34.6 (91)	30.7 (4)	NA	0.726
Partner’s sexual dysfunction (perceived by the patient), % (n)	32.0 (84)°	7.7 (1)	NA	**0.019**
FSFI score				
Desire	2.4 [1.2–3.6]*°	1.2 [1.2–1.8]*	4.2 [3.6–4.8]	** < 0.001**
Arousal	2.7 [1.5–4.2]*	1.3 [1.2–2.3]*	5.1 [4.2–5.4]	** < 0.001**
Lubrication	3.6 [1.5–4.5]*	1.8 [0.7–4.5]*	5.7 [5.3–6.0]	** < 0.001**
Orgasm	3.2 [1.2–4.8]*	1.2 [0.6–4.6]*	5.6 [4.8–6.0]	** < 0.001**
Satisfaction	3.2 [1.6–5.2]*°	2.0 [0.8–2.7]*	5.8 [5.2–6.0]	** < 0.001**
Pain	3.2 [0.8–5.6]*	2.6 [0.4–4.7]*	6.0 [5.2–6.0]	** < 0.001**
Total	19.8 [11.2–25.5]*	10.6 [5.4–19.9]*	30.3 [28.3–33.2]	** < 0.001**
FSDS-R total score	21.0 [9.0–34.2]*	45.5 [22.5–47.7]*	3.0 [0.0–10.0]	** < 0.001**

*P* values refers to statistically significant differences among the 3 groups (or 2 groups, in case of missing data). ° = Significant difference vs. FSD Hyper-PRL, * = Significant difference vs. no-FSD (derived from pairwise comparisons). Bold indicates statistical significance

Data are expressed as mean ± SD when normally distributed, median (quartile) when not normally distributed, and percentage when categorical

*HDL* high-density lipoprotein, *LDL* low-density lipoprotein, *DHEAS* dehydroepiandrosterone sulfate, *FSH* follicle stimulating hormone, *LH* luteinizing hormone, *TSH* thyroid-stimulating hormone, *MHQ* middlesex hospital questionnaire, *BUT* body uneasiness test, *FSFI* female sexual function index, *FSDS-R* female sexual distress scale revised, *PRL* prolactin, *SIS1* sexual inhibition scale (due to threat of performance failure), SIS2 sexual inhibition scale (due to threat of performance consequences), SES sexual excitation scale

**Fig. 1:**
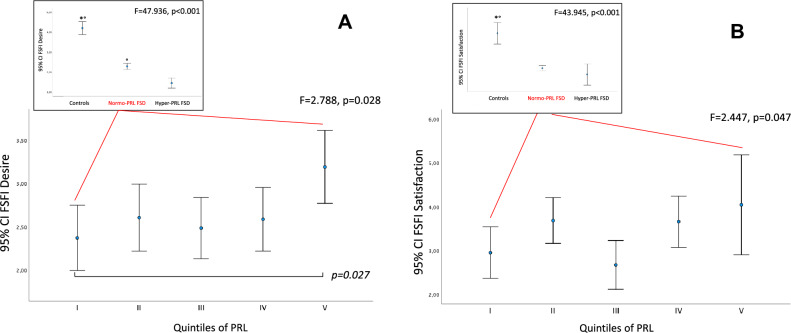
**A** and **B** Differences in Female Sexual Function Index (FSFI) Desire (panel A) and Satisfaction (panel B) domains, in women consulting for sexual symptoms, stratified according to their prolactin (PRL) levels, and controls. Statistic is derived one-way ANOVA and post hoc analysis, performed applying a Bonferroni correction, and general multivariate regression model. Main panel: data adjusted for age, years since menopause, body mass index, and cortisol levels. Inset: data adjusted for partner’s sexual dysfunction, graduation and current use of hormonal replacement therapy. I quintile: PRL 5.12–6.53 µg/L (109–139 mU/L). II quintile: PRL 6.54–8.45 µg/L (140–180 mU/L). III quintile: PRL 8.46–11.03 µg/L (181–235 mU/L). IV quintile: PRL 11.07–14.55 µg/L (238–310 mU/L). V quintile: PRL 14.58–24.59 µg/L (312–523 mU/L). * significantly different from Normo-PRL FSD; ° significantly different from Hyper-PRL FSD. *FSFI* female sexual function index, *FSD* female sexual dysfunction

We then analyzed the differences in terms of FSFI scores (Total and subdomains) in FSD normo-PRL women divided into 5 subgroups according to quintiles of PRL levels. For Desire, a statistically significant difference emerged among the 5 subgroups, after adjusting for confounders (age, years since menopause, body mass index, and cortisol levels) (*F* = 2.788, *p* = 0.028; Fig. 1A). At post hoc analysis, applying a Bonferroni correction, women with PRL in the I quintile (5.12–6.53 µg/L; 109–139 mU/L) reported significantly lower FSFI Desire scores than those with PRL in the V quintile (14.58–24.59 µg/L; 312–523 mU/L) (*p* = 0.027; Fig. 1A). Significant differences were found among the 5 groups also for FSFI Satisfaction (*F* = 2.447, *p* = 0.047), but no two-by-two differences emerged at post hoc analysis (Fig. 1B). In addition, no differences were found among PRL quintiles and the other FSFI domains or the FSDS-R score (not shown). Figure 1A e 1B insets show FSFI Desire (1A) and Satisfaction (1B) scores in FSD normo-PRL women as compared to FSD hyper-PRL and controls.

Subsequently, we investigated, in FSD normo-PRL women, eventual correlations between PRL levels—considered as a continuous variable—and the clinical, biochemical and psychometric parameters investigated (Table 2). At univariate analysis, PRL was negatively associated with age (*p* < 0.0001), menopause (*p* < 0.001), years since menopause (*p* < 0.001), FSH (*p* = 0.001), LH (*p* = 0.016) BMI (*p* = 0.038), total cholesterol (*p* = 0.016), LDL cholesterol (*p* = 0.033), and HbA1c (*p* = 0.025), while positively correlated with morning cortisol (*p* < 0.001), D-4-androstenedione (0.001), DHEAS (*p* = 0.021), and SHBG (*p* = 0.047) (Table 2). However, when adjusted for age, several of these correlations disappeared, and only menopausal status (*p* = 0.013) retained a significant negative, and cortisol a significant positive association with PRL (*p* = 0.007) (Table 2).

**Table 2:** Correlations between prolactin levels and clinical and laboratory parameters at univariate and multivariate analysis, after adjusting for age, in normo-prolactinemic women consulting for sexual symptoms

	Prolactin		*β*	*p*
	*Pearson’s coefficient*	*p*		
Age	− **0.333**	** < 0.001**	–	–
Menopause	− **0.350**	** < 0.001**	− **0.230**	**0.013**
Years since menopause	− **0.281**	** < 0.001**	− 0.096	0.253
Use of psychiatric medications	0.040	0.530	0.003	0.966
Use of hormonal contraception	**0.136**	**0.032**	0.040	0.527
Use of hormonal replacement therapy	0.022	0.735	0.029	0.641
BMI	− **0.131**	**0.038**	− 0.072	0.234
Waist circumference	− 0.120	0.065	− 0.706	0.481
Fasting glycemia	− 0.106	0.105	− 0.045	0.478
HbA1c	− **0.182**	**0.025**	− 0.117	0.137
Total cholesterol	− **0.157**	**0.016**	− 0.032	0.627
Triglycerides	− 0.082	0.212	− 0.053	0.399
HDL cholesterol	0.026	0.689	0.069	0.274
LDL cholesterol	− **0.142**	**0.033**	− 0.039	0.561
Morning cortisol	**0.238**	** < 0.001**	**0.184**	**0.007**
Morning ACTH	0.036	0.876	− 0.087	0.686
TSH	− 0.004	0.955	0.008	0.892
FSH	− **0.210**	**0.001**	− 0.025	0.752
LH	− **0.157**	**0.016**	0.028	0.712
17b-estradiol	0.043	0.603	− 0.003	0.968
D-4-Androstenedione	**0.259**	**0.001**	0.085	0.371
DHEA-S	**0.157**	**0.021**	− 0.043	0.571
Total testosterone	0.025	0.709	− 0.021	0.739
SHBG	**0.144**	**0.047**	0.097	0.155

Bold indicates statistically significant correlations

*BMI* body mass index, *HDL* high-density lipoprotein, *LDL* low-density lipoprotein, *ACTH* Adrenocorticotropic hormone, *TSH* thyroid-stimulating hormone, *FSH* follicle stimulating hormone, *LH* luteinizing hormone, *DHEA-S* dehydroepiandrosterone sulfate, *SHBG* sex hormone binding globulin, *FSFI* = female sexual function index, *FSDS-R* female sexual distress scale-revised

**Fig. 2:**
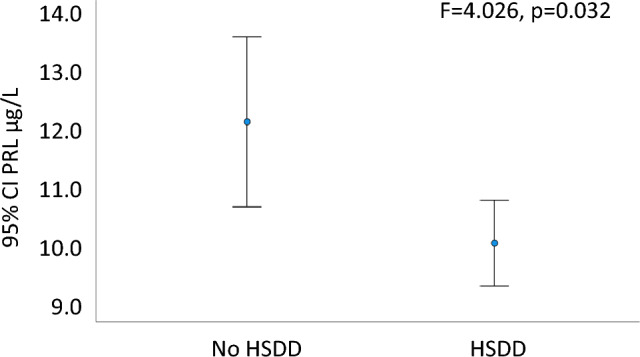
Means and 95% CIs of prolactin (PRL) according to the presence of Hypoactive Sexual Desire Disorder (HSDD). Data were adjusted for years since menopause

**Fig. 3:**
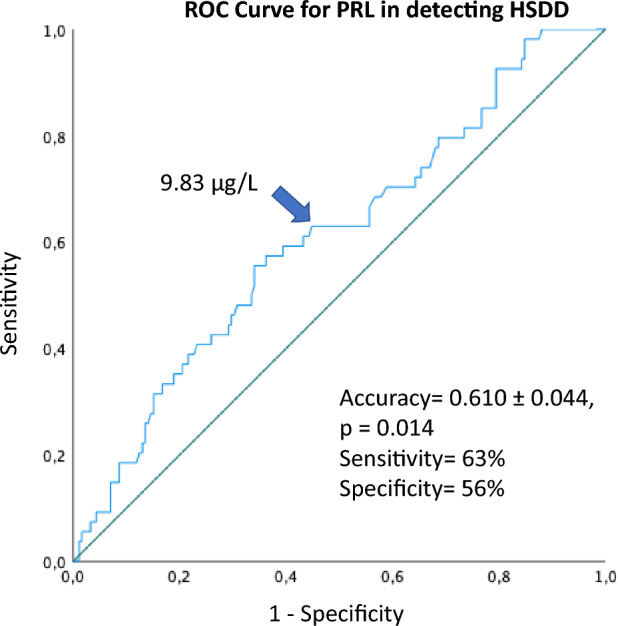
Receiver operating characteristic (ROC) curve for prolactin (PRL) in the identification of Hypoactive Sexual Desire Disorder (HSDD). The arrow indicates the threshold of 9.83 µg/L, identified as the PRL value with the best sensitivity and specificity in detecting HSDD

When we considered the clinical diagnosis of low desire combined with distress (HSDD), we found that women with HSDD presented a significantly lower PRL level than those without, even after adjusting for years since menopause (*F* = 4.026, *p* = 0.032, Fig. 2). Interestingly, a ROC curve analysis for PRL levels showed an accuracy (area under the ROC curve) of 0.610 ± 0.044, *p* = 0.014, in predicting HSDD (Fig. 3). In particular, when a threshold of 9.83 μg/L (209 mU/L) was chosen, sensitivity and specificity for HSDD were 63% and 56%, respectively.

Finally, we compared normoPRL women with PRL levels ≥ and < 9.83 μg/L (Table 3). Subjects with PRL < 9.83 μg/L were older, with higher BMI, WC and SPB values, and presented higher FSH, LH, total and LDL cholesterol levels, but lower cortisol levels (Table 3). When psychological characteristics were considered, subjects with PRL < 9.83 μg/L showed a higher BUT-B PSDI score (mirroring body image uneasiness), and a lower SIS1 score, which indicates lower levels of sexual inhibition due to the threat of performance failure (Table 3). At multivariate analysis, after adjusting for age, only the difference in SIS1 score (*F* = 8.221, *p* = 0.006) and cortisol levels (*F* = 8.815, *p* = 0.003) between women with PRL < 9.83 μg/L vs. women with ≥ 9.83 μg/L retained statistical significance. The difference in SIS1 score was still statistically significant when introducing both age and cortisol levels in the multivariate model (*F* = 6.152, *p* = 0.017).

**Table 3:** Differences in psychosexual, metabolic and hormonal parameters between normoPRL women with PRL levels ≥ and < 190 mU/L

	PRL < 9.83 μg/L (*n* = 140)	PRL ≥ 9.83 μg/L (n = 124)	*p*
Psychosexual parameters			
MHQ total score	36.0 [28.5–46.0]	38.0 [27.0–47.0]	0.665
MHQ A	7.0 [4.0–10.0]	8.0 [5.0–11.0]	0.158
MHQ F	6.0 [4.0–7.0]	6.0 [4.0–8.0]	0.856
MHQ O	7.0 [4.0–9.0]	6.0 [4.0–9.0]	0.982
MHQ S	5.0 [3.0–7.0]	5.0 [2.75–7.25]	0.930
MHQ D	6.0 [4.0–9.0]	5.0 [4.0–9.0]	0.237
MHQ I	5.0 [4.0–7.0]	6.0 [4.0–8.0]	0.224
BUT-A GSI	1.0 [0.5–1.5]	1.0 [0.5–2.0]	0.700
BUT-B PSDI	2.3 [1.8–3.3]	2.0 [1.5–2.6]	**0.041**
BUT-B PST	5.0 [0.0–13.0]	5.5 [0.0–13.5]	0.399
SIS1*	33.5 [27.0–37.0]	36.0 [33.0–42.0]	**0.015**
SIS2	34.0 [29.0–40.0]	35.0 [32.0–39.0]	0.828
SES	39.0 [33.0–47.0]	45.0 [34.0–52.0]	0.161
Metabolic and hormonal parameters			
Age (y)	49.5 ± 12.3	41.1 ± 12.0	** < 0.001**
Years since menopause (y)	5.0 ± 6.1	1.5 ± 3.6	** < 0.001**
BMI (kg/m^2^)	25.9 ± 6.1	24.2 ± 5.7	**0.015**
Waist circumference (cm)	95.4 ± 15.4	91.3 ± 15.9	**0.021**
SBP (mm Hg)	125.0 [110.0–135.0]	120 [110.0–130.0]	**0.009**
DBP (mm Hg)	79.0 [70.0–80.0]	71.0 [70.0 – 80.0]	0.055
Fasting glycemia (mmol/L)	5.0 ± 1.1	4.4 ± 1.1	0.141
Total cholesterol (mmol/L)	5.5 ± 1.1	5.0 ± 0.9	** < 0.001**
Triglycerides (mmol/L)	0.9 [0.7–1.2]	0.8 [0.6 – 1.2]	0.241
HDL cholesterol (mmol/L)	1.6 ± 0.4	1.6 ± 0.4	0.445
LDL cholesterol (mmol/L)	3.4 ± 0.9	2.9 ± 0.8	** < 0.001**
Morning cortisol (nmol/L)	383.5 [302.5–470.7]	448.3 [370.4–546.6]	**0.001**
Morning ACTH (pmol/L)	2.5 [1.6–5.1]	4.2 [3.5–11.9]	**0.047**
TSH (mIU/L)	1.7 [1.1–2.8]	1.8 [1.3–2.4]	0.795
FSH (IU/L)	49.6 [7.3–77.9]	9.1 [6.3–51.5]	**0.002**
LH (IU/L)	22.7 [5.7–34.6]	7.0 [4.8–26.4]	**0.014**
17b-estradiol (pmol/L)	91.9 [52.5–172.5]	128.5 [71.7–191.1]	0.212
D-4-Androstenedione (nmol/L)	3.1 [1.9–5.2]	4.4 [2.7–7.6]	**0.004**
DHEAS (μmol/L)	2.5 [1.5–4.2]	3.0 [1.5–4.3]	0.284
Total testosterone (nmol/L)	0.9 [0.5–1.3]	1.0 [0.5–1.4]	0.167
SHBG (nmol/L)	58.1 [39.4–83.5]	72.3 [50.9–99.7]	**0.006**

Data are expressed as mean ± SD when normally distributed, median (quartile) when not normally distributed, and percentage when categorical

*PRL* prolactin, *MHQ* middlesex hospital questionnaire, *MHQ* *A* free anxiety, *MHQ* *F* phobic anxiety, *MHQ* O obsessive–compulsive traits and symptoms, *MHQ S* somatization anxiety, *MHQ D* depressive symptoms, *MHQ I* = hysteric symptoms, *BUT* = body uneasiness test, *SIS1* = sexual inhibition due to threat of performance failure, *SIS2* = Sexual inhibition due to threat of performance consequences, *SES* = sexual excitation scale, *BMI* = body mass index, *DHEAS* = dehydroepiandrosterone sulfate, *FSH* = follicle stimulating hormone, *HDL* = high-density lipoprotein, *LDL* = low-density lipoprotein, *LH* = luteinizing hormone, *TSH* = thyroid-stimulating hormone

^*^And bold indicate statistically significant difference between the 2 groups (*p* < 0.05)

## Discussion

Our findings indicate that, among FSD women with normo-PRL levels, those with the lowest levels demonstrated a poorer sexual desire than those with PRL in the highest quintile of the normal range. We also confirmed previous evidence, showing that normo-PRL women had significantly higher desire than hyper-PRL women, independently of menopausal status. Intriguingly, PRL levels < 9.83 μg/L in FSD women could predict the presence of HSDD and a lower SIS1 score, which is related to sexual inhibition due the threat of performance failure.

The relationship between PRL levels and sexual function in women is poorly studied. From a neuroendocrine perspective, the secretion of PRL is finely regulated by dopamine, with a negative feedback [32]. Dopamine transmission, in turn, could be affected by the serotonin tone: specifically, it has been theorized that increased serotonin levels may act by reducing dopamine, thus negatively affecting desire [33]. Considering the reported biological effect of PRL on the brain in both genders [4], some studies have demonstrated that PRL levels increase immediately after orgasm (either induced by masturbation or coitus), with a persistent elevated value for at least one hour after sexual activity, both in men and women [34–36]. Therefore, PRL has been traditionally considered as a plasma biomarker of orgasm with potential sexual inhibitory activity during the refractory period [20, 37]. In line with this view, available studies conducted in very small samples of hyperprolactinemic women demonstrated that Total FSFI and all its subdomain scores declined as a function of increased PRL [14, 21, 38]. This relationship was confirmed by an improvement of sexual function when PRL levels were normalized using pharmacological therapy [14]. However, no data are available on the potential role of PRL level on sexual function in normo-PRL women.

Therefore, we conducted the present study, enrolling a large population of normo-PRL FSD women (*n* = 264), and demonstrated that FSD women with a PRL level in the lowest quintile of the normal range were those with the worse sexual profile at FSFI, in particular showing the lowest score in the Desire domain, even after adjusting for confounders. In contrast, no significant differences were observed in lubrication, arousal and pain domains among the quintiles subgroups. These data, although generated using a clinical questionnaire, substantiate the notion that the brain, more than peripheral organs, is the preferential target for PRL activity. Accordingly, even if the PRL receptor has been detected not only in the brain but also in several genital organs, its expression in the vagina is almost scanty [39]. This finding could be considered in contradiction with the known estrogenic-dependency of the PRL system in women. However, the unique and special hormonal regulation of the vagina has been recently recognized by demonstrating that the human distal vagina is robustly regulated not only by estrogens but also androgens [40–42].

Another important finding of our study is that we were able to identify a threshold of PRL level within the normal range which discriminates the presence of HSDD. According to the ROC curve analysis, the predicting PRL level was 9.83 μg/L, with a sensitivity and specificity of 63% and 56%, respectively. Indeed, when dividing our FSD population based on this PRL threshold, we found that women with PRL level < 9.83 μg/L had a lower sexual inhibition due to threat of performance failure (SIS1 score). This PRL threshold also predicted a lower cortisol level. Therefore, considering the known association between PRL and cortisol, a hormone tightly related to a stressful condition [43], we performed the analysis using both of them as confounders of the above-mentioned clinical outcomes. Interestingly, the SIS1 score resulted as being more closely linked to PRL than to cortisol level, the latter losing significant association in the multivariate analysis.

In both genders, PRL excess, specifically when associated with pituitary adenomas, has been associated with a higher risk of MetS, adipose tissue dysfunction index and reduced glucose tolerance, with the normalization of PRL leading to an improved metabolic pattern [44, 45]. On the opposite, in men with sexual complaints, low PRL levels have been associated with a worse metabolic profile [21]. Similar results were observed in women, but no data on their sexual function were reported [46, 47]. In the present study, we confirmed these findings also in women with FSD. Interestingly, we found that low PRL was related to higher BMI, HbA1c, total and LDL cholesterol; however, these associations seem to be mainly modulated by aging. In contrast, metabolic alterations did not alter the association between PRL and FSFI Desire when tested as potential confounders in a multi-adjusted model, together with age, years since menopause and cortisol levels. This observation is in line with previous data showing that metabolic alterations did not exert a detrimental effect on the desire domain in FSD women [48, 49].

Another interesting point is relative to the other extremity of the continuum of sexual desire: hypersexuality. This has been recently defined as “a recurrent lack of control of intense and repetitive sexual impulses, which causes distress or has a clinically significant impact on functioning” [50]. Noteworthy, pathological hypersexuality has been noted at a significantly higher frequency in prolactinoma patients treated with dopamine agonists (DA), and it has been related to DA-induced hypoprolactinemia [51]. However, this effect seems to show a gender-specificity, as hypersexuality has been described only rarely in DA-treated women [23].

Several limitations have to be recognized. The small number of pathological hyper-PRL women enrolled implies that the results should be verified in larger clinical trials. In addition, among hyper-PRL women, 11% were diagnosed with macroprolactinemia at further evaluation. The clinical significance of macroprolactinemia, especially in the context of sexual function, is still poorly understood; however, previous studies suggested a selective impairment in sexual desire in women with macroprolactinemia [22]. Among other limitations, PRL levels were detected on a single blood sample, and only if pathological, repeated as a serial sampling (at 0’ and 30’ minutes); finally, the clinical setting (women consulting for sexual symptoms) limits the generalizability of our findings.

Regarding the low specificity of the proposed cut-off, it should be emphasized that we are not suggesting the use of PRL levels to screen or make a diagnosis of HSDD, neither in the general population, nor in the FSD population, but to draw attention toward a subgroup of patients. Indeed, it’s important to underline that HSDD is an “umbrella term”, covering several medical and psychosocial conditions that could not be systematically reflected by low PRL levels. HSDD should always deserve a biopsychosocial approach, as all other FSD. However, in the future, it would be relevant to investigate whether different levels of baseline sexual inhibition predict different therapeutic outcomes.

The strengths of this research are based on the presence of a control group and on the detection of hormonal levels, which was performed in the early follicular phase for pre-menopausal women. In addition, the diagnostic workflow for the pathological conditions was performed according to the current Endocrine Society guidelines [52]. Another important strength of the study relies on the fact that, at the time of writing, it is the first systematically assessing the role of PRL levels in a large sample of women consulting for FSD.

## Conclusions

Our findings have some clinical relevance, since they highlight the importance of measuring PRL level during the diagnostic workflow of FSD, not only to ascertain the presence of a hyper-PRL condition, but also to potentially predict the presence of HSDD or of a lower inhibitory trait on sexual function when < 9.83 μg/L. Longitudinal data are needed to investigate whether this cut-off could predict better therapeutic outcomes due to a reduced inhibitory profile.

### Supplementary Information

Below is the link to the electronic supplementary material.

Supplementary file1 Differences in Female Sexual Function Index (FSFI) Desire (panels A and C) and Satisfaction (panels B and D) domains, in women consulting for sexual symptoms with normal (normo-PRL FSD) or pathologic PRL levels (hyper-PRL FSD) and controls, stratified according to menopausal status. Statistic is derived one-way ANOVA and post hoc analysis, performed applying a Bonferroni correction, and general multivariate regression model. *= significantly different from Normo-PRL FSD; °= significantly different from Hyper-PRL FSD. FSFI = Female Sexual Function Index. FSD = female sexual dysfunction. (PPTX 7048 KB)
