# Supplementary material for: Low prolactin level identifies hypoactive sexual desire disorder women with a reduced inhibition profile
Source: J Endocrinol Invest. 2023 May 18;46(12):2481–92. doi: 10.1007/s40618-023-02101-8 (PMC10632269; doi:10.1007/s40618-023-02101-8)
Supplement: Supplementary file 1 — Supplementary file1 Differences in Female Sexual Function Index (FSFI) Desire (panels A and C) and Satisfaction (panels B and D) domains, in women consulting for sexual symptoms with normal (normo-PRL FSD) or pathologic PRL levels (hyper-PRL FSD) and controls, stratified according to menopausal status. Statistic is derived one-way ANOVA and post hoc analysis, performed applying a Bonferroni correction, and general multivariate regression model. *= significantly different from Normo-PRL FSD; °= significantly different from Hyper-PRL FSD. FSFI = Female Sexual Function Index. FSD = female sexual dysfunction. (PPTX 7048 KB) [file 40618_2023_2101_MOESM1_ESM.pptx]

## Slide 1
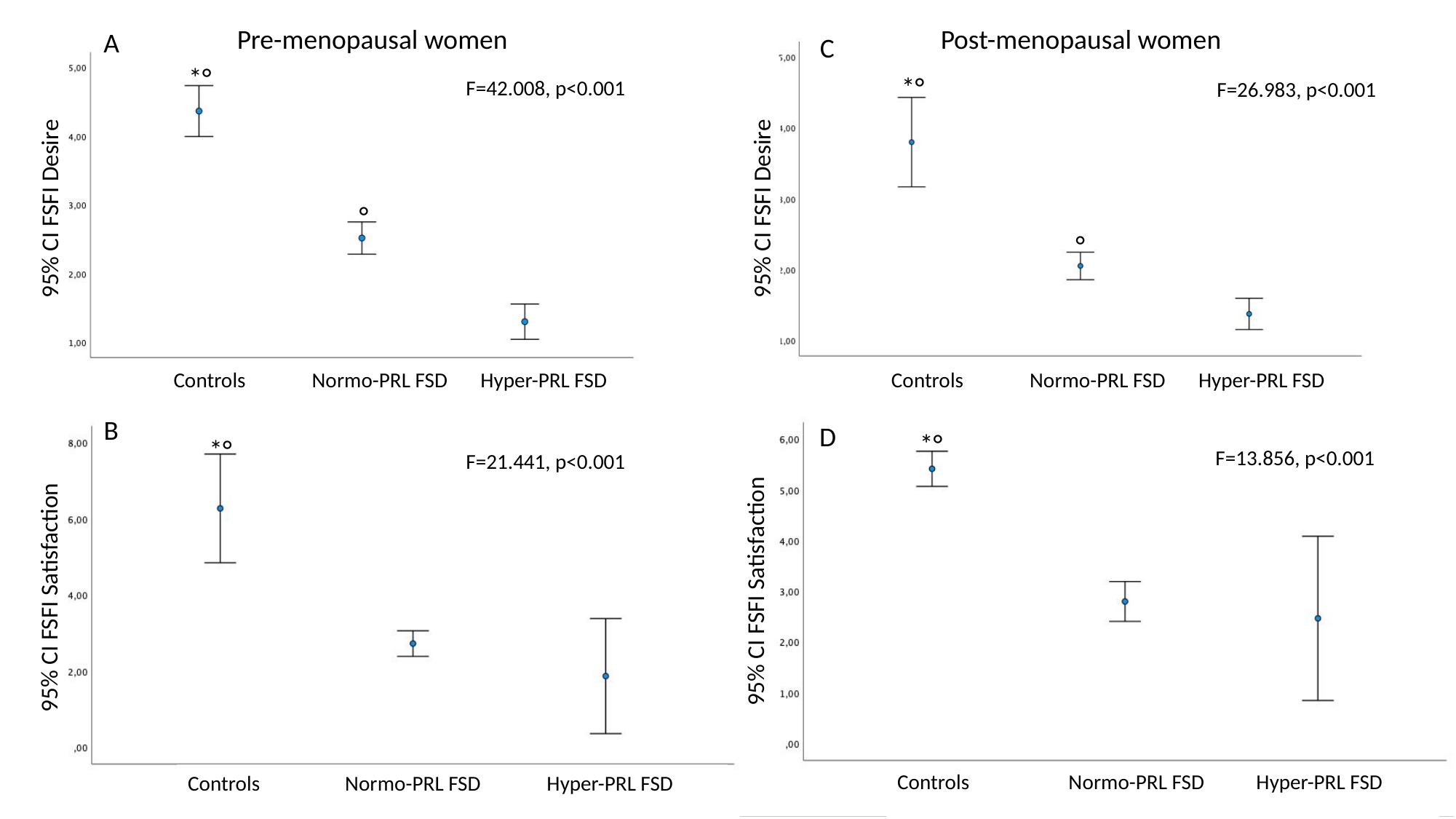

Post-menopausal women
Pre-menopausal women
A
C
*°
*°
F=42.008, p<0.001
F=26.983, p<0.001
95% CI FSFI Desire
95% CI FSFI Desire
°
°
Controls Normo-PRL FSD Hyper-PRL FSD
Controls Normo-PRL FSD Hyper-PRL FSD
Controls Normo-PRL FSD Hyper-PRL FSD
95% CI FSFI Satisfaction
Controls Normo-PRL FSD Hyper-PRL FSD
B
D
*°
*°
F=13.856, p<0.001
F=21.441, p<0.001
95% CI FSFI Satisfaction
Controls Normo-PRL FSD Hyper-PRL FSD
